# Red cell distribution width and its polygenic score in relation to mortality and cardiometabolic outcomes

**DOI:** 10.3389/fcvm.2023.1294218

**Published:** 2023-11-20

**Authors:** Jingxue Pan, Jiangming Sun, Isabel Goncalves, Michael Kessler, Yan Hao, Gunnar Engström

**Affiliations:** ^1^ Division of Child Healthcare, Department of Paediatrics, Tongji Hospital, Tongji Medical College, Huazhong University of Science and Technology, Wuhan, China; ^2^ Department of Clinical Sciences, Lund University, Malmö, Sweden; ^3^ Regeneron Genetics Center, Tarrytown, NY, United States

**Keywords:** red cell distribution width, cardiovascular disease, polygenic score, diabetes, RDW

## Abstract

**Introduction:**

Elevated red cell distribution width (RDW) has been associated with a range of health outcomes. This study aims to examine prognostic and etiological roles of RDW levels, both phenotypic and genetic predisposition, in predicting cardiovascular outcomes, diabetes, chronic kidney disease (CKD) and mortality.

**Methods:**

We studied 27,141 middle-aged adults from the Malmö Diet and Cancer study (MDCS) with a mean follow up of 21 years. RDW was measured with a hematology analyzer on whole blood samples. Polygenic scores for RDW (PGS-RDW) were constructed for each participant using genetic data in MDCS and published summary statistics from genome-wide association study of RDW (*n* = 408,112). Cox proportional hazards regression was used to assess associations between RDW, PGS-RDW and cardiovascular outcomes, diabetes, CKD and mortality, respectively.

**Results:**

PGS-RDW was significantly associated with RDW (Pearson's correlation coefficient = 0.133, *p* < 0.001). RDW was significantly associated with incidence of stroke (hazard ratio (HR) per 1 standard deviatio*n* = 1.06, 95% confidence interval (CI): 1.02–1.10, *p* = 0.003), atrial fibrillation (HR = 1.09, 95% CI: 1.06–1.12, *p* < 0.001), heart failure (HR = 1.13, 95% CI: 1.08–1.19, *p* < 0.001), venous thromboembolism (HR = 1.21, 95% CI: 1.15–1.28, *p* < 0.001), diabetes (HR = 0.87, 95% CI: 0.84–0.90, *p* < 0.001), CKD (HR = 1.08, 95% CI: 1.03–1.13, *p* = 0.004) and all-cause mortality (HR = 1.18, 95% CI: 1.16–1.20, *p* < 0.001). However, PGS-RDW was significantly associated with incidence of diabetes (HR = 0.96, 95% CI: 0.94–0.99, *p* = 0.01), but not with any other tested outcomes.

**Discussion:**

RDW is associated with mortality and incidence of cardiovascular diseases, but a significant association between genetically determined RDW and incident cardiovascular diseases were not observed. However, both RDW and PGS-RDW were inversely associated with incidence of diabetes, suggesting a putative causal relationship. The relationship with incidence of diabetes needs to be further studied.

## Introduction

Red cell distribution width (RDW) is a measure of the heterogeneity of the red cell volumes, which is commonly used for subclassifying different types of anaemia clinically. High RDW has been associated with several adverse health events, such as incidence of stroke (1), coronary events (CE) (2, 3), diabetes (4) and all-cause mortality (5) in general populations. Several possible explanations for the relationship between RDW and health outcomes have been proposed, including inflammation (6), oxidative stress and altered erythrocytes survival (7, 8). Nevertheless, owing to the inherent limitations of observational studies, the causal relationships between RDW and health outcomes remain uncertain.

In the past decades, large-scale genome-wide association studies (GWAS) have identified hundreds of genetic variants that are associated with cardiometabolic diseases (9) and with RDW (10, 11). Based on that, Mendelian randomization (MR) studies are able to conduct examinations of the causal relationship between RDW, venous thromboembolism (VTE) (12) and haemorrhagic strokes (13). However, the application of MR analysis has often been limited to genome-wide significant variants, potentially leading to decreased statistical power due to the relatively small contribution of individual genetic variants in explaining the variance of complex traits (14). On the other hand, polygenic score (PGS), which aggregate the cumulative effects of many genetic variants, is able to capture an increased probability of disease with reasonable statistical power (14–16). PGS is becoming popular in predicting complex diseases such as type-2 diabetes, coronary artery disease and inflammatory bowel disease (17). Association between PGS for RDW and cardiovascular diseases has also been examined in an interesting cross-sectional study suggesting the possibility of using genetic susceptibility to infer causal relationships (18).

Our objective was to investigate the relationship between phenotypically measured and genetically determined RDW and various health outcomes, including diabetes, incidence of chronic kidney disease (CKD), a range of cardiovascular diseases such as atrial fibrillation (AF), stroke, myocardial infarction (MI), heart failure (HF), VTE, and all-cause mortality. We conducted our research using data from a general population study, the Malmö Diet and Cancer study (MDCS, *n* = 27,141) with a follow-up period of up to 27 years.

## Methods

### Study population

The Malmö Diet and Cancer study (MDCS) is a large prospective cohort study from the city of Malmö, Sweden. During 1991–1996, all men born 1923–1945 and women born 1923–1950 living in the city of Malmö were invited in the screening center to participate in the study. The overall participation rate was 40.8% (19). A total of 30,446 participants underwent the baseline examinations. Here, we excluded subjects with missing genotype data, resulting in 28,977 subjects included. Additionally, subjects with missing information on RDW (*n* = 1,820) or outliers of RDW were excluded (*n* = 16). In total, 27,141 subjects were included in the final study sample for analysing associations with incidence of CE, stroke, AF, HF, VTE, CKD, diabetes and mortality. Since diabetes or a CVD event potentially could affect RDW and life span of the red cells (8), we excluded individuals with endpoints before the baseline examination, resulting in slightly different numbers for the various outcomes (range 25,929–27,141) (Supplementary Figure 1: study flow chart for study selection).

A study of representativity and mortality in participants and non-participants has been published. Prevalence of smoking and obesity was comparable in MDCS and another study from the same city—a postal survey with participation rate of 75% (20). However, the mortality rates were lower in MDCS participants than in non-participants, suggesting that MDCS is a comparably healthy cohort (19).

All participants provided and signed written consent and the study conforms to the principles of the Declaration of Helsinki. This study was approved by the Ethical Committee at Lund University (LU 51–90, LU 2009/633, LU 2011/356).

### Baseline examinations and red cell distribution width measurements

The baseline information was collected using extensive questionnaires, blood sample collection and physical examination. Standing height and weight were recorded with light clothing and no shoes. BMI was calculated as weight/height^2^ (kg/m^2^). Non-fasting blood samples were taken during the baseline examinations. Serum and plasma were separated within one hour and stored at −80°C until the analyses. RDW-SD was used in this study, measured in fresh and heparinized blood by a Sysmex K1000 counter (www.sysmex.com). RDW-SD was defined as the width (fL) of the red cell distribution curve at the height of 20% above the baseline (21). Smoking status was ascertained from self-administered questionnaires, which were divided into three groups (regular or occasional smokers, non-smokers and missing). Apolipoprotein A1 (ApoA1) and B (ApoB) were analysed using Quest Diagnostics (San Juan Capistrano, CA). Fasting plasma glucose (FPG) was measured using HemoCue (HemoCue AB, Ängelholm, Sweden). HbA1c was measured by ion exchange chromatography, using the Swedish Mono-S standardization system. Baseline prevalent diabetes was collected by self-reported history of use of anti-diabetic medication or physician's diagnosis of diabetes from questionnaire.

### Genotyping and quality control of the Malmö diet and cancer study

The subjects included in the MDCS were genetically tested using Illumina GSA v1 genotyping array. Prior to imputation, quality controls on the genotyped single nucleotide polymorphism (SNP) were conducted by removing low quality variants [mismatched probe, minor allele frequency (MAF) ≤ 0.01, incorrect assignment of allelic variant, failed genotype calling or *p*-value from Hardy-Weinberg Equilibrium test less than 1 × 10^−15^]. Samples exhibiting evidence of gender mismatch or possessing an overall sample call rate below 90% were excluded from the analysis. Population structure was characterized using principal component analysis (PCA) by implementing the FlashPCA (22) on the genotyped data. Data were imputed with reference panel of Haplotype Reference Consortium (HRC r1.1) using the Michigan Imputation Server (23).

### Polygenic scores (PGS) for RDW

A Bayesian regression framework PRS-continuous shrinkage (PRS-CS) (24) was conducted to build PGS for RDW. Restricted to common variants (MAF > 0.05) that are biallelic, PRS-CS was used to infer posterior effect sizes of SNP which were based on linkage disequilibrium reference panel of HapMap3 in a European sample and GWAS summary statistics on RDW (*n* = 408,112) (25), where age, sex and principal components (PCs) were adjusted in the GWAS analyses. Using the PLINK (version 1.90) (26), PGS for RDW were constructed for 28,977 MDCS samples by aggregating the obtained posterior effect sizes for 1,009,029 SNPs. PGS for RDW is a relative measure in arbitrary units. High PGS for RDW represent high RDW levels.

### Endpoint ascertainment

The outcomes of interest in this study were, incidence of CKD, diabetes, cardiovascular outcomes (including HF, CE, stroke, AF, VTE) and all-cause mortality. All participants were followed from the baseline examinations until the event of interest, emigration, or last follow-up date (31st December 2018), whichever came first. For VTE, the participants were followed up till 31st December 2013.

In this study, information regarding all-cause mortality was retrieved from the Swedish Cause of Death Register (CDR) (27), which covers all deaths among Swedish citizens. Death certificates with information about underlying causes of death were written by a registered physician and coded according to the *International Classification of Diseases*, 9th and 10th revision (ICD-9 and ICD-10) codes.

Information regarding incident CE, stroke, AF, HF, CKD, or VTE were obtained from national patient registers. The Swedish inpatient register has been in operation during the follow-up period and data from this registry has been reported to have acceptable validity for epidemiological research (28). For incident CE, data linkage with the Swedish patient register and the CDR were used to retrieve cases (27, 28). Incidence of CE were defined as fatal or non-fatal MI (ICD-9 code 410 or ICD-10 code I21) or death due to ischemic heart disease (ICD-9 codes 410-414; ICD-10 codes I21-I25).

Stroke was defined as codes 430, 431,434 or 436 (ICD-9), or I60-61 or I63–64 (ICD-10). Ischemic stroke was defined as 434 (ICD-9) or I63 (ICD-10) (28, 29).

The diagnostic code for AF was 427D for ICD-9 and I48 for ICD-10 (30, 31). HF was defined as a primary diagnosis of ICD-9 code 428 or ICD-10 code I50 (32). VTE included deep vein thrombosis (DVT) and pulmonary embolism (PE), which was defined as ICD-9 code: 415B (PE), and 451 (DVT); and ICD-10 code: I26 (PE), and I80 (DVT) as the primary diagnosis (33).

Incident CKD was defined as code: 585-586 (ICD-9) and N18 and N19 (ICD-10). Incidence of CKD was retrieved from Swedish patient register (34) and the Swedish renal registry was searched for any additional CKD cases (35).

Incident diabetes was defined using several registers, including the Malmö HbA1c register, the Swedish national Diabetes register, the Swedish patient register, the CDR and the Swedish drug prescription register which have been described previously (36).

### Statistical analysis

Means ± standard deviations (SDs) were reported for continuous variables and numbers with percentages were reported for categorical variables. The Pearson and Spearman correlation were performed for the correlations of two continuous variables. Cox regression models were implemented to obtain hazard ratios (HR) with 95% confidence interval (CI) per 1-SD increase of RDW and PGS for RDW, respectively. Adjustments were kept to a minimum and limited to a few key confounders known from the literature (Model 1 was crude model; model 2 was additionally adjusted for age, sex, smoking status, BMI, and diabetes). For PGS for RDW, the models were additionally adjusted for the first 5 principal components (PCs) of the population structure, since genetic ancestry may explain associations between variants and a specific phenotype. Interactions between age and sex and RDW or PGS-RDW, respectively, were tested using multiplicative interaction terms in Cox regressions with adjustments in model 2.

A *p* value <0.05 was considered significant. All statistical analyses were carried out using SPSS V.28 (IBM, Armonk, New York, USA).

## Results

### Baseline characteristics of study population

Baseline characteristics across the quartiles of PGS-RDW are shown in Table 1. Across the quartiles of PGS-RDW, we found that there were significant relationships between PGS-RDW and RDW, haemoglobin, Apo A1, and HbA1c. The distribution of RDW and PGS-RDW in this study is illustrated in Supplementary Figures 2, 3. From the figure, we could see that PGS-RDW was normally distributed and associated with RDW in the current study.

**Table 1:** The characteristics of study population across the quartile of PGS-RDW.

	Total	Q1	Q2	Q3	Q4	*p*-for trend
Participants (numbers)	27,141	6,785	6,785	6,786	6,785	
RDW (fL)	40.7 ± 3.40	40.2 ± 3.41	40.6 ± 3.32	40.9 ± 3.40	41.3 ± 3.36	<0.001
PGS-RDW	−0.06 ± 0.46	−0.64 ± 0.24	−0.20 ± 0.09	0.09 ± 0.09	0.52 ± 0.22	<0.001
Age (years)	58.2 ± 7.61	58.3 ± 7.59	58.3 ± 7.63	58.0 ± 7.62	58.2 ± 7.61	0.302
Male sex (*n*, %)	10,692, 39.4	2,707, 39.9	2,629, 38.7	2,620, 38.6	2,736, 40.3	0.106*
BMI (kg/m^2^)	25.7 ± 3.97	25.7 ± 3.97	25.7 ± 3.92	25.7 ± 3.97	25.8 ± 4.03	0.135
Diabetes (*n*, %)	827, 3.0	211, 3.1	214, 3.2	199, 2.9	203, 3.0	0.867*
Smoking (*n*, %)	7,562 (27.9)	1,879 (27.7)	1,844 (27.2)	1,963 (28.9)	1,876 (27.6)	0.263
Hemoglobin (mg/dl)	141.8 ± 12.1	142.7 ± 12.2	141.9 ± 12.1	141.4 ± 12.0	141.1 ± 11.9	<0.001
Anemia (*n*, %)	807 (3.0)	181 (2.7)	178 (2.6)	213 (3.1)	235 (3.5)	0.010
Apo A1 (mg/dl)	156.8 ± 28.2	155.8 ± 27.6	156.9 ± 28.3	157.4 ± 28.6	157.2 ± 28.3	0.008
Apo B (mg/dl)	107.2 ± 26.1	107.3 ± 26.0	107.2 ± 26.0	107.7 ± 25.9	106.5 ± 26.4	0.066
HbA1c (%, *n* = 5,189)	4.8 (4.5, 5.1)	4.7 (4.4, 5.0)	4.8 (4.5, 5.1)	4.8 (4.5, 5.1)	4.9 (4.6, 5.2)	<0.001
Glucose (mmol/L, *n* = 5,193)	4.9 (4.6, 5.3)	4.9 (4.6, 5.3)	4.9 (4.6, 5.3)	4.9 (4.6, 5.3)	4.9 (4.6, 5.3)	0.379

The results were presented as means ± standard deviations for continuous variable and numbers, percentages for categorical variables.

Q, quartile; RDW, red cell distribution width; PGS, polygenic score; BMI, body mass index; Apo A1, Apolipoprotein A1; Apo B, Apolipoprotein B; HbA1c, hemoglobin A1c.

* The results were obtained by Chi-Square.

The correlation coefficients between RDW and PGS-RDW, respectively, and other risk factors are presented in Table 2. The Pearson's correlation of PGS-RDW and RDW were 0.133, *p* < 0.001. RDW was significantly correlated to age, BMI, HbA1c and glucose. There was a significant correlation between PGS-RDW and HbA1c (*r* = 0.101, *p* < 0.001, *n* = 5,186) and BMI (*r* = 0.014, *p* = 0.024), but no significant correlation with fasting glucose (*r* = 0.003, *p* = 0.808).

**Table 2:** The correlations between PGS-RDW, RDW and several factors.

Variables		RDW	Age	BMI	Hemoglobin	Apo A1	Apo B	HbA1c	Glucose
Numbers		*n* = 27,141	*n* = 27,141	*n* = 27,099	*n* = 27,007	*n* = 26,659	*n* = 26,656	*n* = 5,186	*n* = 5,193
PGS-RDW	R1	0.133	−0.004	0.014	−0.059	0.013	−0.012	0.101	0.003
	P	<0.001	0.461	0.024	<0.001	0.028	0.044	<0.001	0.808
	R2	0.135	−0.005	0.013	−0.054	0.016	−0.012	0.149	−0.013
	P	<0.001	0.368	0.035	<0.001	0.011	0.058	<0.001	0.353
RDW	R1	–	0.118	−0.118	−0.053	0.121	−0.066	0.051	−0.088
	P	–	<0.001	<0.001	0.001	<0.001	<0.001	<0.001	<0.001
	R2	–	0.112	−0.124	−0.054	0.123	−0.066	0.190	−0.071
	P	–	<0.001	<0.001	<0.001	<0.001	<0.001	<0.001	<0.001

R_1_, Pearson's correlation coefficient; R_2_, Spearman's correlation coefficient; RDW, red cell distribution width; PGS, polygenic score; BMI, body mass index; Apo A1, Apolipoprotein A1; Apo B, Apolipoprotein B; HbA1c, hemoglobin A1c.

### Cardiometabolic outcomes predicted by PGS-RDW and RDW

**Figure 1:**
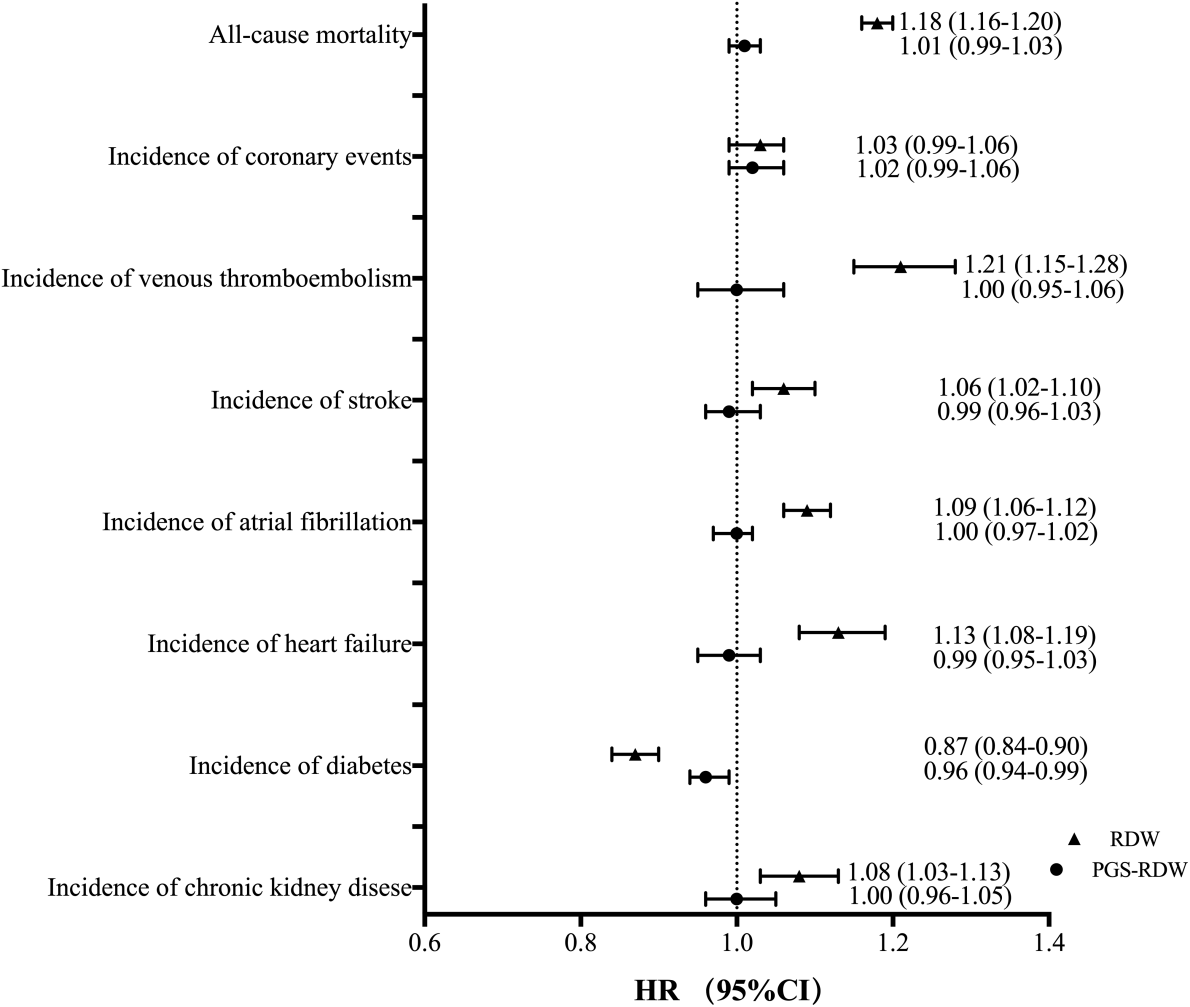
The forest plot presenting relationships between RDW and PGS of RDW and mortality and cardiometabolic diseases. The model was adjusted for age, sex, BMI, smoking and diabetes. For PGS-RDW and mortality and cardiometabolic disease, the model was additionally adjusted for PC1-PC5. RDW, red cell distribution width; PGS, polygenic score; HR, hazard ratio; CI, confidence interval; CE, coronary events; AF, atrial fibrillation; CKD, chronic kidney disease; HF, heart failure; VTE, venous thromboembolism.

Elevated RDW was significantly associated with incidence of stroke, AF, HF, VTE, CKD and all-cause mortality. These relationships remained significant after additional adjustments for risk factors (age, sex, smoking status, BMI, and diabetes) (Table 3 and Figure 1). RDW was inversely associated with incidence of diabetes (adjusted HR: 0.87, 95% CI: 0.84–0.90, *p* < 0.001).

**Table 3:** The associations between PGS-RDW, RDW and mortality and cardiometabolic outcomes in the MDCS.

Outcomes (events/ individuals, *n*/*n*)	Per 1 SD increase of PGS-RDW^a^	Per 1 SD increase of RDW^b^
All-cause mortality (11,179/27,141)		
HR (95% CI) Model 1	1.01 (0.99–1.03)	1.31 (1.29–1.33)
HR (95% CI) Model 2	1.01 (0.99–1.03)	1.18 (1.16–1.20)
Incidence of CE (3,384/26,621)		
HR (95% CI) Model 1	1.02 (0.99–1.06)	1.10 (1.07–1.14)
HR (95% CI) Model 2	1.02 (0.99–1.06)	1.03 (0.99–1.06)
Incidence of stroke (3,124/26,846)		
HR (95% CI) Model 1	0.99 (0.96–1.03)	1.15 (1.11–1.19)
HR (95% CI) Model 2	0.99 (0.96–1.03)	1.06 (1.02–1.10)
Incidence of AF (5,195/26,867)		
HR (95% CI) Model 1	1.00 (0.97–1.03)	1.12 (1.09–1.15)
HR (95% CI) Model 2	1.00 (0.97–1.02)	1.09 (1.06–1.12)
Incidence of CKD (1,761/27,134)		
HR (95% CI) Model 1	1.00 (0.96–1.05)	1.10 (1.04–1.15)
HR (95% CI) Model 2	1.00 (0.96–1.05)	1.08 (1.03–1.13)
Incidence of HF (1,992/27,062)		
HR (95% CI) Model 1	0.99 (0.95–1.03)	1.21 (1.16–1.26)
HR (95% CI) Model 2	0.99 (0.95–1.03)	1.13 (1.08–1.19)
Incidence of diabetes (4,427/25,929)		
HR (95% CI) Model 1	0.97 (0.94–1.00)	0.85 (0.82–0.88)
HR (95% CI) Model 2	0.96 (0.94–0.99)	0.87 (0.84–0.90)
Incidence of VTE (1,332/26,943)		
HR (95% CI) Model 1	1.00 (0.95–1.06)	1.23 (1.17–1.30)
HR (95% CI) Model 2	1.00 (0.95–1.06)	1.21 (1.15–1.28)

RDW, red cell distribution width; PGS, polygenic score; HR, hazard ratio; CI, confidence interval; CE, coronary events; AF, atrial fibrillation; CKD, chronic kidney disease; HF, heart failure; VTE, venous thromboembolism.

^a^ Model 1 was adjusted for PC1-5; Model 2 was additionally adjusted for age, sex, BMI, smoking and diabetes.

^b^ Model 1 was crude model; Model 2 was additionally adjusted for age, sex, BMI, smoking and diabetes.

For PGS-RDW, we found an inverse association with incidence of diabetes [adjusted HR: 0.96 (95% CI: 0.94–0.99), *p* = 0.011 in model 2] (adjusted for age, sex, PCs 1–5, smoking status, BMI) (Table 3 and Figure 1). PGS-RDW was not significantly associated with stroke, AF, VTE, HF, CKD or mortality after full adjustments (Table 3 and Figure 1).

The sex-specific relationships between RDW, PGS-RDW and outcomes are presented in Supplementary Table 1. There was a significant interaction between RDW and sex with respect to mortality, indicating a stronger relationship in men. For incidence of diabetes, the relationship with RDW was stronger in women (Supplementary Table 1). We also tested the age-specific relationships between RDW, PGS-RDW and outcomes. There were significant interactions between RDW and age with respect to mortality and incidence of diabetes. The associations with mortality were stronger in individuals aged <60 years, while the reduced risk of diabetes was stronger in individuals aged ≥60 years (Supplementary Table 2).

### Sensitivity analyses

As PGS-RDW was significantly associated with HbA1c, we further examined the relationships between PGS-RDW and incidence of diabetes after excluding diabetes cases whose diabetes diagnosis was based on high HbA1c values in the HbA1c register. The results showed that RDW and PGS-RDW were still significantly associated with incident diabetes: adjusted HR per 1 SD increase in PGS-RDW: 0.96, 95% CI: 0.92–0.99, *p* = 0.013; HR per 1 SD increase in RDW: 0.89, 95% CI: 0.85–0.92, *p* < 0.001. As anaemia is a risk factor for RDW and cardiometabolic disease, we also additionally adjusted for anaemia in the last models of associations between RDW, PGS-RDW and cardiometabolic diseases, but the results were almost unchanged (data not shown).

## Discussion

RDW has been related to risk of cardiometabolic diseases and mortality in several previous studies (1, 4, 5), but possible etiological relations between RDW and cardiometabolic diseases remain unclear. We found significant relationships between RDW and incidence of several adverse events, such as incidence of CKD, CVDs and all-cause mortality in the MDCS. Interestingly, with exception of diabetes, PGS-RDW was not significantly associated with incidence of these outcomes. For incidence of diabetes, there was a significant inverse association with both RDW and PGS-RDW after adjustments for potential risk factors. The results show that RDW is an unspecific risk marker for various adverse health outcomes but is probably not causally related to mortality and CVD. However, the nature of the inverse relationship between RDW and incidence of diabetes is of interest and warrants further studies.

Several observational studies have found that RDW is independently associated with CVDs, including MI (2), stroke (1, 37), AF (38), HF (39), VTE (40) and mortality (41, 42). The present results of RDW confirm and expand on previous results from MDCS, as well as other cohort studies. Several potential mechanisms were proposed to contribute to these associations, such as inflammation (6) and oxidative stress (7), which could affect red blood cell (RBC) production and erythropoiesis, and thereby RDW. There are also some previous studies of genetically predicted-RDW in relation to CVDs (12, 18, 43, 44). A study from the UK Biobank reported that genetically increased RDW was not predictive for incident cardiovascular disease. Furthermore, genetic risk score of LDL and genetic risk score of systolic blood pressure were inversely associated with RDW. The authors suggested that relationship between RDW and cardiovascular outcomes may partly be related to aging (43). Another previous study (18) showed non-significant relationships between genetically-increased-RDW and CVDs. In accordance with the previous studies, we did not find any significant relationship between PGS-RDW and CVDs in this study with individual follow-up data. This suggests that although RDW could be a valuable marker of predicting incident of CVDs, it is probably not causally related to increased CVD or mortality risks.

Inflammation or oxidative stress have been reported to increase RDW, as inflammation might be related to reduced RBC formation (6). A previous study (45) reported that the low response to erythropoietin treatment in patients with low-grade inflammation could lead to low red cell turn-over. As inflammation is commonly seen in many diseases, including CVDs, it is presumable that this might lead to high RDW in individuals. Besides, a previous study of the associations between common demographic and clinical characteristics (including laboratory tests) with variability in RDW, indicated that only small percentage of the observed variation in RDW was explained by routinely assessed clinical or laboratory variables [including inflammation markers, such as white blood cells (WBC), etc.] (46). Also, our previous studies reported RDW was associated with mortality and abdominal aortic aneurysm after multiple adjustments of routine laboratory parameters (i.e., WBC, etc.) (5, 47). Hence, other routine laboratory parameters do not seem to fully explain the relationships between RDW and mortality or cardiovascular outcomes. The relationships between RDW and outcomes might be driven by many factors including inflammatory factors. In addition, it is also possible that unmeasured confounders could explain the associations between RDW and all-cause mortality and major cardiovascular events in observational studies. More research is needed in this regard.

RDW has previously been linked to adverse outcomes in patients with CKD or with impaired kidney function (48–50). We observed significant relationships between RDW and incidence of CKD in the MDCS, however, we did not find any significant relationship between PGS-RDW and incident CKD and the results suggest that RDW could be a biomarker for CKD risk in the general population, but probably not a causal factor for incident CKD.

The inverse relationship between RDW and diabetes is in accordance with a previous study from the present cohort, but in contrast to results from a Chinese study (51). In our study we also found a significant inverse relationship between PGS-RDW and incidence of diabetes. The relationship between RDW and diabetes is complex. First, many studies have shown positive relationship between RDW and HbA1c (52), similarly to our findings. In our study we also found a significant relationship between HbA1c and PGS-RDW. This is probably explained by a relationship between RDW and increased life span of the circulating red cells (8). Longer red cell lifespan will result in a higher proportion of red cells being exposed to glucose for long time (4). Individual differences in erythrocyte lifespan can be large enough to explain variations in HbA1c (53). However, in our study, the relationship between PGS-RDW and incidence of diabetes persisted in a sensitivity analysis when we excluded diabetes cases who were identified using the register of HbA1c measurements. Another important factor is that hyperglycaemia might affect erythrocytes in several aspects (54), such as increased volumes, reduced deformability and osmotic stability of the red cells (55), which might lead to shorter red cell life span and higher concentrations of reticulocytes. Hence, presence of hyperglycaemia could potentially modify the relationships between RDW and glucose or HbA1c. The present study of an initially non-diabetic population found significant inverse relationships with incidence of diabetes, both for RDW and PGS-RDW. The results are supported by results from UK Biobank reporting an inverse relationship between the genetic risk scores for type 2 diabetes and RDW, although this became non-significant after adjustment for multiple comparisons (43). Nevertheless, the relationship between RDW and diabetes need to be further studied also with other types of study design.

## Strengths and limitations

The large population-based cohort study with individual follow-up data is a major strength of this study. The PGS-RDW was constructed using about one million genetic polymorphisms, which should increase the statistical power. However, even though we used a large cohort with individual follow-up data, low statistical power remains a potential problem in Mendelian randomization studies.

The GWAS study we used to calculate the PGS-RDW, and the subjects of the present study were mostly from European ancestry (44). This limits the generalizability to subjects from non-European ancestry. Furthermore, the GWAS used to calculate PGS-RDW was based on RDW-CV, and not RDW-SD, which was used as measure of RDW in this cohort. However, RDW and PGS-RDW were also significantly correlated in this study. Another limitation is potential horizontal pleiotropic effects. Especially for diabetes, more studies are needed to examine whether there are any causal relationships.

## Conclusions

RDW is associated with mortality and increased incidence of CKD and different CVDs, but the non-significant relationship with PGS-RDW indicates that the relationships are not causal. However, both RDW and PGS-RDW were inversely associated with incidence of diabetes, indicating a potentially causal association. RDW is not causally involved in most CVDs but could still be a useful marker. The relationship with incidence of diabetes needs to be further studied.

## Data Availability

The data sets presented in this article are not publicly available because the data is owned by Lund University, and the research is coordinated by the steering committee of MDCS. Applications for research data can be addressed to Anders Dahlin, data manager, email: anders.dahlin@med.lu.se. Legal restrictions to data access may apply.

## Regeneron Genetics Center

Members included in Regeneron Genetics Center are listed below:

**RGC Management & Leadership Team**

Regeneron Genetics Center, Tarrytown, NY, USA; Gonçalo Abecasis, D. Phil., Adolfo Ferrando, M.D., Ph.D., Aris Baras, M.D., Michael Cantor, M.D., Giovanni Coppola, M.D., Andrew Deubler, M.P.S., Aris Economides, Ph.D., Katia Karalis, Ph.D., Luca A. Lotta, M.D., Ph.D., Lyndon J. Mitnaul, Ph.D., John D. Overton, Ph.D., Jeffrey G. Reid, Ph.D., Alan Shuldiner, M.D., Katherine Siminovitch, M.D.

Contribution: All authors contributed to securing funding, study design and oversight. All authors reviewed the final version of the manuscript.

**Sequencing & Lab Operations**

Regeneron Genetics Center, Tarrytown, NY, USA: John D. Overton, Ph.D., Christina Beechert, Erin D. Brian, Laura M. Cremona, Ph.D., Hang Du, Caitlin Forsythe, M.S., Zhenhua Gu, M.S., Kristy Guevara, M.S., Michael Lattari, Alexander Lopez, M.S., Kia Manoochehri, Manasi Pradhan, M.S., Raymond Reynoso, Ricardo Schiavo, Maria Sotiropoulos Padilla, M.S., Chenggu Wang, M.S., Sarah E. Wolf, M.S.

Contribution: Performed and are responsible for sample genotyping and exome sequencing, conceived and are responsible for laboratory automation, and responsible for sample tracking and the library information management system.

**Clinical Informatics**

Regeneron Genetics Center, Tarrytown, NY, USA: Michael Cantor M.D., Amelia Averitt, Ph.D., Nilanjana Banerjee, Ph.D., Dadong Li, Ph.D., Sameer Malhotra, M.D., Justin Mower, Ph.D., Mudasar Sarwar, Deepika Sharma, Ph.D., Jeffrey C. Staples, Ph.D., Jay Sundaram, Sean Yu, Ph.D., Aaron Zhang, Ph.D.

Contribution: Development and validation of clinical phenotypes used to identify study participants and (when applicable) controls.

**Genome Informatics & Data Engineering**

Regeneron Genetics Center, Tarrytown, NY, USA: Jeffrey G. Reid, Ph.D., Mona Nafde, M.S., George Mitra, Sujit Gokhale, Andrew Bunyea, Janice Clauer, M.S., MBA, Krishna Pawan Punuru, M.S., Sanjay Sreeram, Gisu Eom, Sujit Gokhale, Benjamin Sultan, M.S., Rouel Lanche, Vrushali Mahajan, Eliot Austin, Koteswararao Makkena, M.S., Sean O'Keeffe, Ph.D., Razvan Panea, Ph.D., Tommy Polanco, Ayesha Rasool, M.S., William J. Salerno, Ph.D., Xiaodong Bai, Ph.D., Lance Zhang, M.S., Boris Boutkov, Ph.D., Evan Edelstein, Alexander Gorovits, Ph.D., Ju Guan, Ph.D., Lukas Habegger, Ph.D., Alicia Hawes, Olga Krasheninina, M.S., Adam J. Mansfield, Evan K. Maxwell, Ph.D., Suganthi Balasubramanian, Ph.D., Suying Bao, Ph.D., Kathie Sun, Ph.D., Chuanyi Zhang, Ph.D.

Contribution: Performed and are responsible for analysis needed to produce exome and genotype data, provided compute infrastructure development and operational support, provided variant and gene annotations and their functional interpretation of variants, and conceived and are responsible for creating, developing, and deploying analysis platforms and computational methods for analyzing genomic data.

**Analytical Genetics and Data Science**

Regeneron Genetics Center, Tarrytown, NY, USA: Gonçalo Abecasis, D. Phil., Manuel Allen Revez Ferreira, Ph.D., Joshua Backman, Ph.D., Kathy Burch, Ph.D., Adrian Campos, Ph.D., Lei Chen, Ph.D., Sam Choi, Ph.D., Amy Damask, Ph.D., Liron Ganel, Ph.D., Sheila Gaynor, Ph.D., Benjamin Geraghty, Ph.D., Akropravo Ghosh, M.S., Salvador Romero Martinez, Christopher Gillies, Ph.D., Lauren Gurski, Joseph Herman, D. Phil., Eric Jorgenson, Ph.D., Tyler Joseph, Ph.D., Michael Kessler, Ph.D., Jack Kosmicki, Ph.D., Nan Lin, Ph.D., Adam Locke, Ph.D., Priyanka Nakka, Ph.D., Jonathan Marchini, Ph.D., Anthony Marcketta, M.S., Joelle Mbatchou, Ph.D., Arden Moscati, Ph.D., Aditeya Pandey, Ph.D., Anita Pandit, M.S., Charles Paulding, Ph.D., Jonathan Ross, Carlo Sidore, Ph.D., Eli Stahl, Ph.D., Maria Suciu, Ph.D., Timothy Thornton, Ph.D., Peter VandeHaar, M.S., Sailaja Vedantam, Ph.D., Scott Vrieze, Ph.D., Rujin Wang, Ph.D., Kuan-Han Wu, Ph.D., Bin Ye, Ph.D., Blair Zhang, Ph.D., Andrey Ziyatdinov, Ph.D., Yuxin Zou, Ph.D., Olivier Delaneau, Ph.D., Maya Ghoussaini, Ph.D., Jingning Zhang, Ph.D.

Contribution: Development of statistical analysis plans. QC of genotype and phenotype files and generation of analysis ready datasets. Development of statistical genetics pipelines and tools and use thereof in generation of the association results. QC, review and interpretation of results. Generation and formatting of results for manuscript figures.

**Therapeutic Area Genetics**

Regeneron Genetics Center, Tarrytown, NY, USA: Katherine Siminovitch, M.D., Brian Hobbs, M.D., Jon Silver, Ph.D., William Palmer, Ph.D., Rita Guerreiro, Ph.D., Jan Freudenberg, M.D., Amit Joshi, Ph.D., Antoine Baldassari, Ph.D., Luca A. Lotta, M.D., Ph.D., Cristen Willer, D. Phil., Sarah Graham, Ph.D., Jonas Bille Nielsen, Ph.D., Mary Hass, Ph.D., Niek Verwei, Ph.D., George Hindy, Ph.D., Jonas Bovijn, M.D., Tanima De, Ph.D., Parsa Akbari, Ph.D., Luanluan Sun, Ph.D., Olukayode Sosina, Ph.D., Arthur Gilly, Ph.D., Peter Dombos, Ph.D., Alan Shuldiner, M.D., Juan Rodriguez-Flores, Ph.D., Moeen Riaz, Ph.D., Manav Kapoor, Ph.D., Gannie Tzoneva, Ph.D., Momodou W. Jallow, Ph.D., Anna Alkelai, Ph.D., Giovanni Coppola, M.D., Ariane Ayer, Veera Rajagopal, M.D., Sahar Gelfman, Ph.D., Vijay Kumar, Ph.D., Jacqueline Otto, Ph.D., Neel Parikshak, M.D., Aysegul Guvenek, Ph.D., Jose Bras, Ph.D., Adolfo Ferrando, M.D., Ph.D., Silvia Alvarez, Ph.D., Jessie Brown, Ph.D., Jin He, Ph.D., Hossein Khiabanian, Ph.D.

Contribution: Development of study design and analysis plans. Development and QC of phenotype definitions. QC, review, and interpretation of association results.

**Research Program Management & Strategic Initiatives**

Regeneron Genetics Center, Tarrytown, NY, USA: Marcus B. Jones, Ph.D., Esteban Chen, M.S., Jaimee Hernandez, M.S., Michelle G. LeBlanc, Ph.D., Jason Mighty, Ph.D., Nirupama Nishtala, Ph.D., Nadia Rana, Ph.D., Jennifer Rico-Varela, Ph.D.

Contribution: Contributed to the management and coordination of all research activities, planning and execution, managed the review of the project.
