# Supplementary material for: Red cell distribution width and its polygenic score in relation to mortality and cardiometabolic outcomes
Source: Front Cardiovasc Med. 2023 Nov 20;10:1294218. doi: 10.3389/fcvm.2023.1294218 (PMC10694461; doi:10.3389/fcvm.2023.1294218)

***Supplementary materials***

**Supplementary table 1. The associations between PGS-RDW, RDW and mortality and cardiometabolic outcomes in men and women, and p-values for interaction with sex**

| **Outcomes (events/individuals, n/n)** | **Per 1 SD increase of PGS-RDW in men *** | **Per 1 SD increase of PGS-RDW in women *** | **Per 1 SD increase of RDW in men #** | **Per 1 SD increase of RDW in women #** |
| --- | --- | --- | --- | --- |
| All-cause mortality (11179/27141) | (5455/10692) | (5695/16449) | (5455/10692) | (5695/16449) |
| HR (95%CI) Model 2 | 1.01 (0.98-1.03), p=0.572 | 1.01 (0.99-1.04), p=0.305 | 1.21 (1.18-1.24), p<0.001 | 1.15 (1.12-1.19), p<0.001 |
| P for interaction with sex | p=0.751 | | p=0.036 | |
| Incidence of CE (3384/26621) | (1932/10273) | (1443/16348) | (1932/10273) | (1443/16348) |
| HR (95%CI) Model 2 | 1.05 (1.00-1.09), p=0.05 | 0.99(0.94-1.04), p=0.664 | 1.05 (1.00-1.10), p=0.044 | 1.01 (0.96-1.07), p=0.631 |
| P for interaction with sex | p=0.108 | | p=0.682 | |
| Incidence of stroke (3124/26846) | (1496/10509) | (1624/16337) | (1496/10509) | (1624/16337) |
| HR (95%CI) Model 2 | 1.00 (0.95-1.05), p=0.906 | 0.99 (0.94-1.04), p=0.697 | 1.07 (1.02-1.13), p=0.010 | 1.05 (0.999-1.11), p=0.054 |
| P for interaction with sex | p=0.938 | | p=0.728 | |
| Incidence of AF (5195/26867) | (2528/10515) | (2659/16352) | (2528/10515) | (2659/16352) |
| HR (95%CI) Model 2 | 0.99 (0.95-1.03), p=0.576 | 1.00 (0.97-1.04), p=0.897 | 1.11 (1.06-1.15), p<0.001 | 1.08 (1.04-1.13), p<0.001 |
| P for interaction with sex | p=0.658 | | p=0.866 | |
| Incidence of CKD (1761/27134) | (1023/10690) | (736/16444) | (1023/10690) | (736/16444) |
| HR (95%CI) Model 2 | 0.98 (0.92-1.04), p=0.463 | 1.04 (0.97-1.12), p=0.298 | 1.10 (1.03-1.18), p=0.003 | 1.02 (0.94-1.11), p=0.629 |
| P for interaction with sex | p=0.165 | | p=0.093 | |
| Incidence of HF (1992/27062) | (1039/10640) | (950/16422) | (1039/10640) | (950/16422) |
| HR (95%CI) Model 2 | 0.98 (0.93-1.04), p=0.540 | 0.997 (0.94-1.06), p=0.938 | 1.19 (1.12-1.26), p<0.001 | 1.07 (1.00-1.15), p=0.05 |
| P for interaction with sex | p=0.729 | | p=0.106 | |
| Incidence of diabetes (4427/25929) | (2091/10054) | (2327/15875) | (2091/10054) | (2327/15875) |
| HR (95%CI) Model 2 | 0.97 (0.93-1.01), p=0.159 | 0.96 (0.92-0.996), p=0.031 | 0.93 (0.89-0.98), p=0.004 | 0.81 (0.77-0.85), p<0.001 |
| P for interaction with sex | p=0.661 | | p=0.001 | |
| Incidence of VTE (1332/26943) | (543/10594) | (788/16349) | (543/10594) | (788/16349) |
| HR (95%CI) Model 2 | 0.996 (0.92-1.08), p=0.933 | 1.00 (0.93-1.07), p=0.979 | 1.23 (1.13-1.33), p<0.001 | 1.22 (1.13-1.31), p<0.001 |
| P for interaction with sex | p=0.911 | | p=0.488 | |

* Model 2 was adjusted for PCs1-5, age, sex, BMI, smoking and diabetes.

# Model 2 was adjusted for age, sex, BMI, smoking and diabetes.

Abbreviations: RDW: red cell distribution width; PGS: polygenic score; HR: hazard ratio; CI: confidence interval; CE: coronary events; AF: atrial fibrillation; CKD: chronic kidney disease; HF: heart failure; VTE: venous thromboembolism.

**Supplementary table 2. The associations between PGS-RDW, RDW and mortality and cardiometabolic outcomes in participants aged <60 years and** **≥60 years, and p-values for interaction with age**

| **Outcomes (events/individuals, n/n)** | **Per 1 SD increase of PGS-RDW in participants aged <60 years *** | **Per 1 SD increase of PGS-RDW in participants aged ≥60 years *** | **Per 1 SD increase of RDW in participants aged <60 years #** | **Per 1 SD increase of RDW in participants aged ≥60 years #** |
| --- | --- | --- | --- | --- |
| All-cause mortality (11179/27141) | (3318/15193) | (7832/11948) | (3318/15193) | (7832/11948) |
| HR (95%CI) Model 2 | 1.01 (0.97-1.04), p=0.701 | 1.01 (0.99-1.03), p=0.453 | 1.29 (1.24-1.33), p<0.001 | 1.22 (1.20-1.25), p<0.001 |
| P for interaction with age | p=0.926 | | p=0.002 | |
| Incidence of CE (3384/26621) | (1270/15053) | (2105/11568) | (1270/15053) | (2105/11568) |
| HR (95%CI) Model 2 | 1.04 (0.99-1.10), p=0.124 | 1.01(0.96-1.05), p=0.794 | 1.08 (1.02-1.14), p=0.013 | 1.07 (1.02-1.11), p=0.005 |
| P for interaction with age | p=0.292 | | p=0.864 | |
| Incidence of stroke (3124/26846) | (1112/15099) | (2008/11747) | (1112/15099) | (2008/11747) |
| HR (95%CI) Model 2 | 1.02 (0.96-1.08), p=0.608 | 0.98 (0.94-1.02), p=0.372 | 1.14 (1.08-1.22), p<0.001 | 1.08 (1.03-1.13), p<0.001 |
| P for interaction with age | p=0.346 | | p=0.188 | |
| Incidence of AF (5195/26867) | (2010/15121) | (3177/11746) | (2010/15121) | (3177/11746) |
| HR (95%CI) Model 2 | 1.01 (0.96-1.05), p=0.837 | 0.99 (0.96-1.03), p=0.583 | 1.11 (1.06-1.16), p<0.001 | 1.14 (1.10-1.18), p<0.001 |
| P for interaction with age | p=0.617 | | p=0.081 | |
| Incidence of CKD (1761/27134) | (635/15189) | (1124/11945) | (635/15189) | (1124/11945) |
| HR (95%CI) Model 2 | 0.98 (0.91-1.06), p=0.620 | 1.01 (0.96-1.07), p=0.654 | 1.11 (1.02-1.20), p=0.016 | 1.12 (1.05-1.19), p<0.001 |
| P for interaction with age | p=0.590 | | p=0.818 | |
| Incidence of HF (1992/27062) | (562/15176) | (1427/11886) | (562/15176) | (1427/11886) |
| HR (95%CI) Model 2 | 1.00 (0.92-1.08), p=0.988 | 0.98 (0.93-1.03), p=0.489 | 1.26 (1.16-1.37), p<0.001 | 1.17 (1.11-1.23), p<0.001 |
| P for interaction with age | p=0.726 | | p=0.145 | |
| Incidence of diabetes (4427/25929) | (2602/14733) | (1816/11196) | (2602/14733) | (1816/11196) |
| HR (95%CI) Model 2 | 0.96 (0.92-0.99), p=0.022 | 0.97 (0.93-1.02), p=0.203 | 0.90 (0.86-0.94), p<0.001 | 0.84 (0.79-0.88), p<0.001 |
| P for interaction with age | p=0.581 | | p=0.031 | |
| Incidence of VTE (1332/26943) | (568/15117) | (763/11826) | (568/15117) | (763/11826) |
| HR (95%CI) Model 2 | 0.96 (0.88-1.04), p=0.266 | 1.04 (0.96-1.11), p=0.341 | 1.26 (1.16-1.37), p<0.001 | 1.23 (1.14-1.32), p<0.001 |
| P for interaction with age | p=0.155 | | p=0.485 | |

* Model 2 was adjusted for PCs1-5, age, sex, BMI, smoking and diabetes.

# Model 2 was adjusted for age, sex, BMI, smoking and diabetes.

Abbreviations: RDW: red cell distribution width; PGS: polygenic score; HR: hazard ratio; CI: confidence interval; CE: coronary events; AF: atrial fibrillation; CKD: chronic kidney disease; HF: heart failure; VTE: venous thromboembolism.

**Supplementary figure 1: study flow chart for study selection.**

**
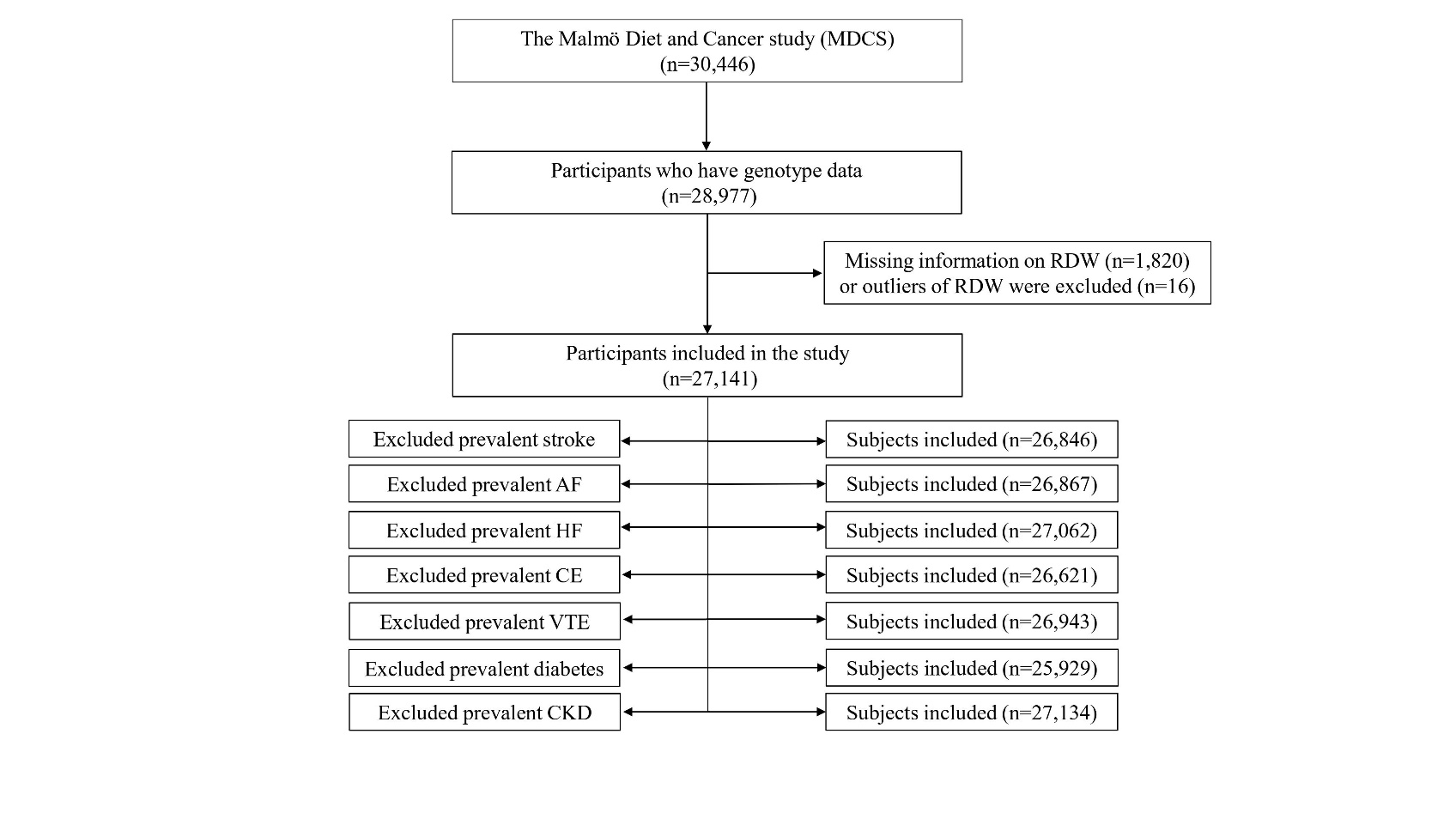
**

**Supplementary figure 2: The histogram of PGS-RDW in the MDCS.**


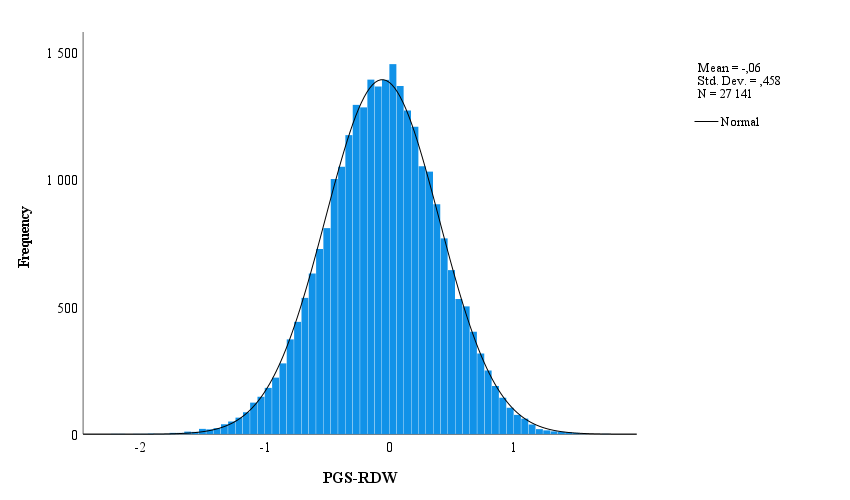


**Supplementary figure 3:** **The scatter plot of RDW and PGS-RDW in the MDCS.**


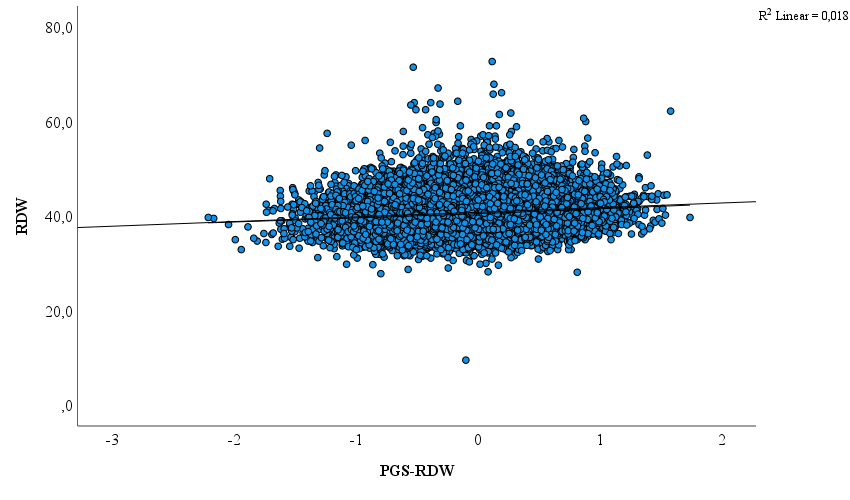

Supplement: Supplementary file 1 [file Datasheet1.docx]
